# Children with Hirschsprung disease in a developing country: A cohort study of the predictors of a positive rectal biopsy result

**DOI:** 10.1097/MD.0000000000031601

**Published:** 2022-11-18

**Authors:** Raed Al-Taher, Hebah Tawfiq Daradkeh, Hiba Hadadin, Abdelrahman Obiedat, Yazan Hijazein, Laith Hijazein, Sarah Obiedat, Yazeed Hadadin, Abdel rahman Al Manasra, Hamza Alduraidi, Malik Juweid

**Affiliations:** a Department of General Surgery, School of Medicine, University of Jordan, Amman, Jordan; b Department of General Surgery, School of Medicine, Jordan University of Science and Technology, Irbid, Jordan; c Department of Pathology, School of Medicine, University of Jordan, Amman, Jordan; d Department of General Surgery, School of Medicine, October 6th University, Cairo, Egypt; e Community Health Nursing Department, School of Nursing, University of Jordan, Amman, Jordan; f Department of Radiology and Nuclear Medicine, School of Medicine, University of Jordan, Amman, Jordan.

**Keywords:** aganglionosis, constipation, Hirschsprung, rectal biopsy

## Abstract

Chronic constipation, which may be habitually or pathologically caused, is one of the most common complaints in children. One of the important pathological causes is Hirschsprung’s disease (HD), which is diagnosed via multiple modalities, mainly rectal biopsy. Our aim was to compare the presentation and different predictive factors for positive rectal biopsy results in a developing country in the Middle East, such as Jordan. This cohort study was conducted at the Jordan University Hospital (JUH). All consecutive children aged <14 years who presented with refractory constipation and underwent rectal biopsies between January 2014 and December 2019 were retrospectively enrolled in the study. In the entire cohort study, 79 patients were enrolled: 45 (57%) were males and 34 (43%) were females. Regarding the biopsy results, 51 (64.6%) cases of refractory constipation without HD and 28 (35.4%) patients with refractory constipation with HD were diagnosed with open rectal biopsies. The male-to-female ratio of HD patients was 3:1. Moreover, 3 (10.7%) children who passed the meconium within the first 24 to 48 hours showed features of HD, while 17 (60.7%) children with delayed passage of the meconium showed features of HD. Abdominal distension was found to be a positive predictor of positive biopsy results (odds ratio [OR] = 4.09, *P* = .011), and soiling was found to be a negative predictor of positive biopsy results (OR = 0.07, *P* = .024). In developing countries, children presenting with HD seem to have similar symptoms and signs to those observed with traditional sampling and staining techniques.

## 1. Introduction

Chronic constipation is one of the most common complaints among children. Most of the time, it is habitual constipation; however, it can be pathological in less common situations. The difficulty in diagnosing pathological causes relies on the multifactorial process that occurs, leading to constipation.

Of the wide spectrum of causes of constipation, Hirschsprung’s disease remains the most alarming, leading to alterations in defecation. Hence, Hirschsprung disease (HD) should be ruled out in patients with chronic constipation.^[1,2]^ Children may present with varying complaints and different presentations according to age, length of the aganglionic segment, and other comorbidities that may be associated with HD.^[2,3]^ Unfortunately, patients usually undergo lengthy procedures and images before the right diagnosis is finally reached.

Rectal biopsies were the most sensitive method for diagnosing HD. However, it is invasive, expensive, and requires a large amount of tissue to represent the faulty colonic segment.^[4]^ To acquire a beneficial sample, two techniques are adopted: the suction and cut technique, and the open sample technique.^[5]^ Regardless of the technique used, biopsies must contain mucosal and submucosal tissue at least 2 cm above the dentate line so that the ganglionic segment will be included in the extracted sample to rule out HD.^[6,7]^

In this study, we aimed to analyze the results of rectal biopsies obtained from children with chronic constipation and whether the information extracted from these biopsies was conclusive, representative, and beneficial for the diagnosis of HD.

## 2. Methodology

### 2.1. Study design and population

This cohort study was conducted at the Jordan University Hospital (JUH) in February 2020. Eligibility criteria included consecutive children aged <14 years, who presented with refractory constipation, and from whom biopsies were taken between January 2014 and December 2019. Patients were retrospectively enrolled and all individuals with missing data were excluded from the study. The total number of patients was 128; however, 19 patients did not have chronic constipation and biopsies were taken for other purposes; 30 had missing data and were thus excluded from the study.

### 2.2. Measured variables

Patient demographics (age, sex, height, and weight), clinical presentation (failure to pass meconium, vomiting, abdominal distention, failure to thrive, abdominal pain, bloody stool, and feeding intolerance), neonatal history (mode of delivery, neonatal intensive care unit (NICU) admission, and congenital anomalies), family history, investigations, biopsy results, and site(s) of aganglionosis were evaluated. Data were extracted from the electronic medical records of the JUH and the Emergency department registries and recorded using Microsoft Excel.

### 2.3. Biopsy technique and processing

All rectal biopsies enrolled in this study were acquired using the open technique or the endoscopic punch method. Suction rectal biopsies were not available at the center at the time of data collection. These samples were received 2 cm above the dentate line in infants (a little higher in older children, up to 4–5 cm from the dentate line), followed by a second biopsy taken proximal to the first sample. In the case of an inconclusive diagnosis, repeated full-thickness biopsies under general anesthesia were performed to acquire a more informative sample.

In an open rectal biopsy, the children were placed in the lithotomy position under general anesthesia, followed by emptying the rectum using rectal washouts. Prophylactic antibiotics were administered before starting the procedure. A full-thickness tissue biopsy specimen was cut (ensuring that the mucosa and submucosa were removed from the muscularis propria) and sent for histopathology.

Samples were processed using hematoxylin and eosin (H&E) to stain formalin-fixed, paraffin-embedded tissue sections in the JUH Pathology Lab. Rectal mucosa and submucosa acetylcholinesterase enzyme (AChE) staining was used to distinguish the presence and absence of ganglionic cells. While there is new evidence to support the importance of studying calretinin receptors, only a few samples have been processed for calretinin staining, as this method was introduced to our center in 2019. Thus, samples processed with calretinin staining were stated, but not analyzed or compared with the other samples to eliminate biases.

### 2.4. Data analysis

Descriptive statistics were calculated using Statistical Package for the Social Sciences version 25.0. Statistical significance was set at *P* < .05. Chi-square tests were performed to determine the relationship between the independent and dependent variables (i.e., the biopsy result). Normal regression tests were also performed to determine the relationship between common signs and symptoms and biopsy results. The study is reported adhering to the Strengthening the Reporting of Observational Studies in Epidemiology statement on reporting of cohort studies.

### 2.5. Ethical consideration

Ethical approval for this study was granted by the Institutional Review Board of the University of Jordan. All participants were informed about the study, and consent was obtained from all participants before enrollment in the study. All procedures contributing to this study complied with the principles of the Helsinki Declaration of 1975 as revised in 2008.

## 3. Results

**Figure 1.**
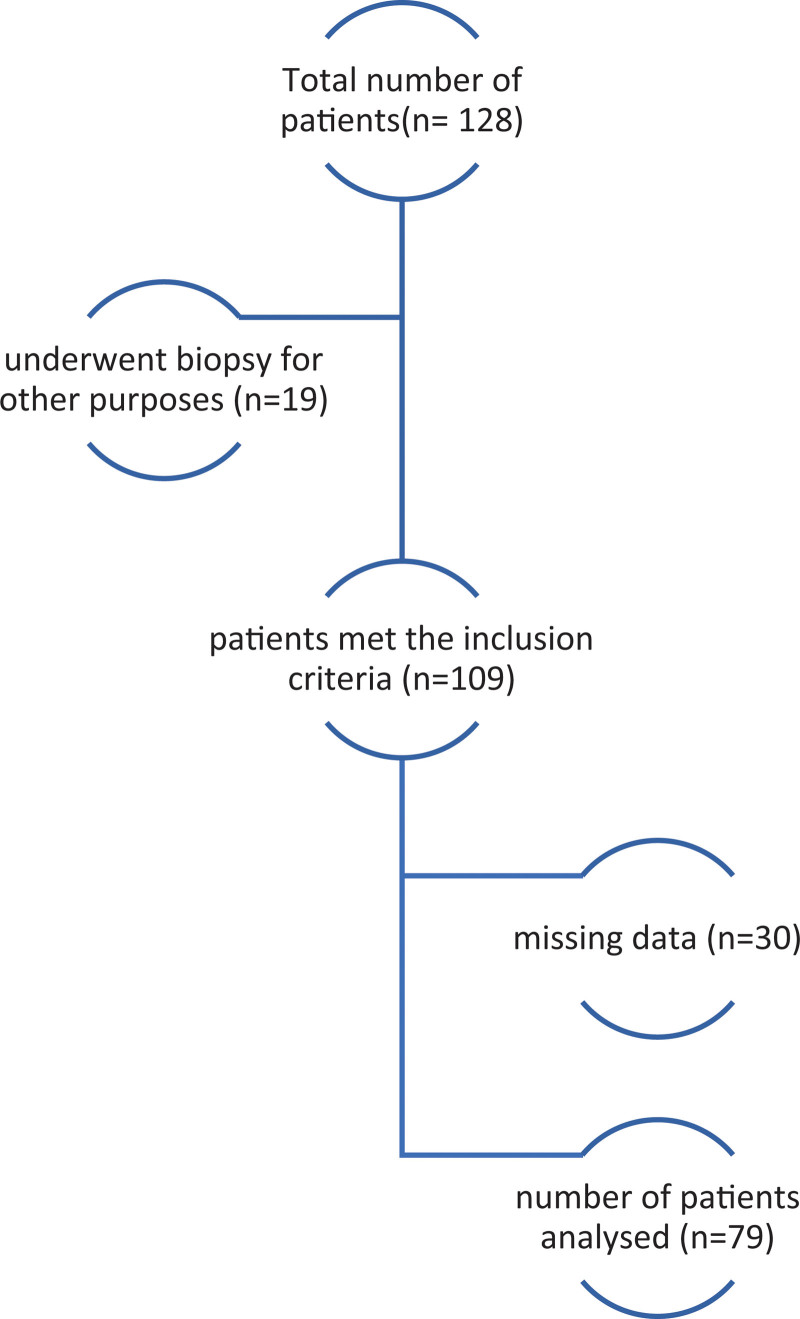
Figure showing the inclusion and exclusion criteria.

The total number of patients was 128; however, 19 patients did not have chronic constipation and biopsies were taken for other purposes; 30 had missing data and thus were excluded from the study (Fig. 1). Only 79 patients met the inclusion criteria, of whom 45 (57%) were males and 34 (43%) were females, and rectal biopsies were taken in part of the workup of refractory constipation. Mode of delivery showed that 40 patients (50.6%) had been delivered vaginally, and 24 patients (30.4%) had been delivered by Cesarean section (CS). Table 1 shows the demographics and predictive factors for positive rectal biopsies.

**Table 1:** Demographics and predicting factors for positive rectal biopsies.

		Refractory constipation		Total, n (%)	*P* value
		Without HD, n (%)	With HD, n (%)		
Gender	Female	27 (52.9)	7 (25)	34 (43)	.019
	Male	24 (47.1)	21 (75)	45 (57)	
Mode of delivery	Normal vaginal delivery	31 (60.8)	9 (32.1)	40 (50.6)	.015
	C-section	11 (21.6)	13 (46.4)	24 (30.4)	
	Missing	9 (17.6)	6 (21.4)	15 (19)	
Weight at birth (kg)	<1.5 (very low)	2 (3.9)	0 (0)	2 (2.5)	.565
	1.5-2.4 (low)	6 (11.8)	3 (10.7)	9 (11.4)	
	≥2.5 (normal)	33 (64.7)	19 (67.9)	52 (65.8)	
	Missing	10 (19.6)	6 (21.4)	16 (20.3)	
Gestational age (wk)	<37 (preterm)	11 (21.6)	3 (10.7)	14 (17.7)	.343
	37–42 (term)	30 (58.8)	19 (67.9)	49 (62)	
	Missing	10 (19.6)	6 (21.4)	16 (20.3)	
NICU admission	Yes	27 (52.9)	17 (60.7)	44 (55.7)	.421
	No	16 (31.4)	6 (21.4)	22 (27.8)	
	Missing	8 (15.7)	5 (17.9)	13 (16.5)	
Passage of meconium	Within 1st 24–48 h of life	23 (45.1)	3 (10.7)	26 (32.9)	.002
	Delayed passage	17 (33.3)	17 (60.7)	34 (43)	
	Missing	11 (21.6)	8 (28.6)	19 (24.1)	
Age at time of rectal biopsy	Birth - 1 mo	17 (33.3)	8 (28.6)	25 (31.6)	.003
	1–6 mo	4 (7.8)	6 (21.4)	10 (12.7)	
	6 mo–1 yr	1 (2)	3 (10.7)	4 (5.1)	
	1–6 yr	19 (37.3)	2 (7.1)	21 (26.6)	
	6–14 yr	10 (19.6)	3 (10.7)	13 (16.5)	
	Missing	0 (0)	6 (21.4)	6 (7.6)	
Consanguinity of parents	Yes	15 (29.4)	7 (25)	22 (27.8)	1
	No	19 (37.3)	9 (32.1)	28 (35.4)	
	Missing	17 (33.3)	12 (42.9)	29 (36.7)	
Family history of HD	Yes	11 (21.6)	6 (21.4)	17 (21.5)	1
	No	25 (49)	13 (46.4)	38 (48.1)	
	Missing	15 (29.4)	9 (32.1)	24 (30.4)	
Contrast enema	Positive result	10 (19.6)	18 (64.3)	28 (35.4)	.001
	Negative result	34 (66.7)	8 (28.6)	42 (53.2)	
	Missing	7 (13.7)	2 (7.1)	9 (11.4)	
Technique of biopsy	Endoscopic	22 (43.1)	11 (39.3)	33 (41.8)	.814
	Open transanal	29 (56.9)	17 (60.7)	46 (58.2)	
Total		51 (100)	28 (100)	79 (100)	

The weight at birth showed that neonates with a birth weight of <1.5 kg, between 1.5 and 2.5 kg, and more than 2.5 kg were 2 (2.5%), 9 (11.4%), 52 (65.8%), respectively. The gestational age showed that 14 (17.7%) of the study population were delivered preterm with a gestational age <37 weeks, and 49 (62%) were delivered between 37 and 42 weeks. NICU admission reports showed that 44 (55.7%) participants had been admitted, and 22 (27.8%) had no NICU admission. Regarding the duration of NICU admission, 37 (46.8%) newborns were admitted 1–30 days, 5 (6.3%) were admitted 31 to 60 days, and 2 (2.5%) were admitted 61 to 100 days. The NICU admission data showed that 2 (4.5%) neonates were admitted due to persistent vomiting, 3 (6.8%) due to abnormal colon/rectum, 10 (22.7%) due to delayed passage of meconium, 6 (13.6%) due to anteriorly displaced anus, 3 (6.8%) due to intestinal obstruction, 4 (9%) due to low birth weight, and 12 (27.3%) due to neonatal jaundice and/or respiratory distress syndrome.

Regarding biopsy results, there were 51 (64.6%) cases of refractory constipation without HD and 28 (35.4%) cases of refractory constipation with HD. The male-to-female ratio of HD patients was 3:1.

Positive histopathological features of HD were observed in 13 (46.4%) biopsies taken from children who had been delivered via CS and 9 (32.1%) biopsies taken from children delivered via normal vaginal delivery (*P* = .015). Moreover, 3 (10.7%) children who passed the meconium within the first 24 to 48 hours showed features of HD, while 17 (60.7%) children with delayed passage of the meconium showed features of HD (*P* = .002).

Histopathological patterns of HD were observed in 3 (10.7%) low birth weight and 19 (67.9%) normal birth weight infants (*P* = .565). Histopathological patterns of HD were also observed in 3 (10.7%) preterm children and 19 (67.9%) term children (*P* = .343). Six (21.4%) children with a family history of HD and 13 (46.4%) with no traceable family history of HD had positive features of HD on biopsy (*P* = 1.000). Seven (25%) children with consanguineous parents had HD (*P* = 1.000).

Regarding biopsy technique, 11 (39.3%) endoscopic and 17 (60.7%) open transanal biopsies yielded specimens with positive features of HD (*P* = .814). Seventy of the 79 enrolled children underwent a contrast enema. Twenty-eight patients had a positive contrast enema, 18 (64.3%) of whom showed features of HD on biopsy. In contrast, 42 children had negative contrast enema, 8 (19%) of whom showed positive features of HD on biopsy.

In terms of presenting symptoms, positive biopsy features were observed in 18 (64.3%) children with abdominal distension (*P* = .089), 9 (32.1%) with vomiting (*P* = .273), 4 (14.3%) with abdominal pain (*P* = .252), 3 (10.7%) with anal stenosis on examination (*P* = .133), 2 (7.1%) with failure to thrive (*P* = .700), 2 (7.1%) with bloody stool (*P* = .701), and 2 (7.1%) with feeding intolerance (*P* = .192). However, only 1 child (3.6%) with soiling had positive HD features on biopsy (*P* = .024).

Congenital anomalies were observed in 15 patients (18.9%). HD was found in 6 (40%) patients: 2 had liver dysfunction, 2 had Down syndrome, 1 had hydrocephaly, and 1 had celiac disease. However, HD was observed in 20 (31.3%) patients with no congenital anomalies (*P* = .406).

Different HD features were observed in the biopsies. Of the 79 children’s biopsies, 28 showed absence of ganglion cells in the submucosal and/or myenteric plexuses. Of these, 9 (32.1%) biopsies showed hypertrophied nerve trunks, and one (3.6%) showed increased AChE activity in the submucosa. However, 11 (39.3%) biopsies showed aganglionis alone, without other features.

Regarding the histopathological extent of aganglionosis retrieved after surgical treatment, 15 (53.5%) biopsies showed aganglionosis in the rectum only, 5 (17.8%) biopsies showed aganglionosis reaching the sigmoid colon, 7 (25%) biopsies showed aganglionosis involving the rest of the colon, and 1 (3.6%) biopsy showed aganglionosis extending to the small bowel.

The likelihood ratio tests showed that abdominal distension and anal tightness on examination were statistically significant (*P* = .005 and *P* = .045, respectively) in predicting the dependent variable (i.e., positive result of HD).

Binomial logistic regression was performed to ascertain the effects of sex, weight at birth, gestational age, NICU admission, delayed passage of meconium, age at time of rectal biopsy, consanguinity, family history of HD, contrast enema result, and biopsy technique on the likelihood that participants had a positive rectal biopsy result with features of HD. One standardized residual with a value of 3.317 standard deviations was retained in the analysis. The logistic regression model was statistically significant, χ^2^ (10) = 23.09, *P* < .010). The model explained 53.7% (Nagelkerke R^2^) of the variance in biopsy results and correctly classified 81.3% of the cases. The sensitivity, specificity, positive predictive value, and negative predictive value were 60%, 90.9%, 75%, and 83.3%, respectively. Of the ten predictor variables, only one was statistically significant: delayed passage of the meconium. Table 2 shows the logistic regression results predicting the likelihood of a positive biopsy result based on multiple predictive factors.

**Table 2:** Logistic regression predicting likelihood of a positive biopsy result based on multiple predicting factors.

	*B*	S.E.	Wald	*df*	*P* value	Odds ratio	95% C.I. for odds ratio	
							Lower	Upper
Gender	2.05	1.09	3.49	1	.062	7.73	0.91	66.02
Weight at birth (kg)	1.88	1.16	2.64	1	.104	6.55	0.68	63.24
Gestational age (wk)	1.85	1.27	2.13	1	.144	6.38	0.53	76.71
NICU admission	1.44	1.15	1.56	1	.212	4.20	0.44	39.95
Passage of meconium	3.16	1.33	5.69	1	.017	23.64	1.76	317.93
Age at time of biopsy	0.12	0.34	0.12	1	.734	1.12	0.57	2.20
Contrast enema	−1.31	0.76	2.95	1	.086	0.27	0.06	1.20
Consanguinity of parents	0.74	0.97	0.58	1	.447	2.09	0.31	13.97
Family history of HD	−0.05	0.95	0.00	1	.957	0.95	0.15	6.06
Technique of biopsy	−0.62	1.07	0.34	1	.562	0.54	0.07	4.36

NICU = neonatal intensive care unit.

Binary logistic regression was conducted to identify the signs and symptoms that predict a positive rectal biopsy result. Logistic regression assumptions, including the assumption of multicollinearity, were tested prior to conducting the model. Positive rectal biopsy results were entered as dependent outcomes, whereas the signs and symptoms of abdominal distension, vomiting, failure to thrive, anal stenosis, and soiling were entered as independent factors. The model was statistically significant, χ^2^ (5) = 17.96, *P* = .003). The model was able to explain 28% (Nagelkerke R^2^) of the variance in rectal biopsy results and correctly classified 73.4% of the cases. Of the 5 independent factors in the model, two showed statistical significance: abdominal distension (B = 1.408, *P* = .011), where cases with abdominal distension had approximately 4 times the odds of showing a positive rectal biopsy, and soiling (B = −2.692, *P* = .024), where cases with soiling had approximately 0.07 the odds of having a positive rectal biopsy. The remaining factors were not statistically significant.

## 4. Discussion

The biopsy results in the JUH showed that the positive features of HD in males were triple those in females, and this difference was statistically significant (*P* = .019). Male predominance was observed among the patients at a tertiary hospital in Bahrain, with a male-to-female ratio of 2.6:1.^[8]^ This finding has also been reported in many other studies.^[9–12]^

JUH is a public teaching hospital and major hospital in Jordan. Jordan has a population of >10 million, 33.05% of whom are children below the age of 15 years (Central Intelligence Agency (2021). Jordan - The World Factbook. Website: https://www.cia.gov/the-world-factbook/countries/jordan/ [accessed 12 Dec 2021]).

In the present study, positive features of HD were observed more frequently in infants born via CS than in those born vaginally (*P* = .015). This is supported by a study conducted by Tateishi et al, who showed that infants born vaginally passed the meconium earlier than those delivered via CS, since the former were more acidotic.^[13]^ Another study conducted in Nigeria supports this result.^[14]^

Our results showed that 50% of children with delayed passage of the meconium had clinically significant features of HD (*P* = .002). In another study, delayed passage of the meconium was shown to be the most important symptom in neonates with HD. Over 90% of HD patients fail to pass the meconium within 24 hours after birth.^[15]^

The relationship between birth weight and the histopathological patterns of HD in this study was not significant (*P* = .565). A study conducted by Arnoldi et al showed that 81% of low birth weight infants showed a delay in the passage of the meconium.^[16]^

Our study showed that 21.4% of preterm and 38.8% of term children had histopathological patterns of HD (*P* = .343). However, there is growing evidence for an association between HD and preterm birth.^[17]^

The classic clinical symptoms of HD include abdominal distension (>90%) and vomiting (>85%), which may be bilious, and failure to pass the meconium during the first 24 hours of life (>60%).^[19,20]^ However, our study showed that abdominal distention, vomiting, and failure to pass the meconium were present in 64%, 32%, and 61% of children with HD, respectively. However, these values were not statistically significant. Our study also showed that soiling is significantly associated with the absence of HD.

Our study did not find a significant relationship between family history and HD. Moreover, consanguinity among parents in the Middle East is quite common; therefore, we studied its relationship with HD, and the results showed no significant differences between them. Furthermore, one study showed that most HD patients did not have a family history of the disease. However, there was a change of 1% for the child to have the disease if one of the parents had it. Another study showed a linkage and association between HD and familial genetic disorders.^[20]^

The results of our study revealed that more than half of the patients who received a negative contrast enema did not have the disease. However, a lesser percentage has been shown between a positive contrast enema and having the disease, but it is still a significant value. This was also the case in a study conducted in South Africa that showed the significance of contrast enema in the diagnosis of HD.^[21]^ A study conducted in Germany showed that contrast enema is a good but not optimal diagnostic technique.^[22]^ Another study showed that contrast enema may show inconclusive results, and that biopsies should be performed.^[23]^

The definitive diagnosis of HD is based on submucosal rectal biopsy. This is performed by one of two procedures: endoscopic biopsy or open transanal under general anesthesia.^[24]^ Occasionally, an open transanal procedure is performed after endoscopic biopsy because it has more tissue present than endoscopic biopsy. However, both the procedures yielded reliable results.^[25]^ Endoscopic biopsy was performed in 33 patients and open transanal biopsy in 46 patients.

Hypertrophied submucosal nerve trunks (≥40 µm) are associated with abnormal nerves and/or aganglionosis.^[5]^ Among the 28 patients without ganglionic cells, 9 (32.1%) had hypertrophied nerve trunks.

Another feature to look for in histopathology is increased AChE activity in the submucosa and mucosa, yet it is not reliable in detecting aganglionosis in the transition zones. Transmural biopsies are the ultimate tool.^[26]^ Only 1 (3.6%) patient with HD showed increased AChE activity.

Finally, in the Middle East, consanguineous marriage is quite frequent; thus, a future study might be conducted on a wider base to include cases from all regional communities to investigate the genetic predisposition of HD and the importance of consanguinity.

## 5. Conclusion

HD patients remain challenging to diagnose; however, clinical presentation has the best predictive value for positive histopathology testing. Although the limitations of sampling and staining remain in terms of easier diagnosis, traditional methods provide comparable and conclusive results. Finally, delayed passage of meconium and abdominal distention were found to be positive predictors of HD, while soiling was found to be a negative predictor of HD.

## 6. Limitations

Our study was conducted retrospectively; thus, data collection was performed using archived handwritten files. In 2017, JUH was in a transition phase from hard copy formatting of data into a fully computerized system. All these challenges have caused some samples to be excluded from the study due to missing data.

All rectal biopsies were performed using the open technique because of the lack of instrumentation to perform the suction technique, which limited the procedures to be more invasive and under general anesthesia in all cases.

The COVID-19 era has delayed the progress of data collection and the paper for an entire year.

Calretinin staining was introduced in 2019; therefore, most of the biopsies processed previously were only stained with hematoxylin and eosin for AChE. This might have increased the sensitivity in the diagnosis of aganglionic tissue if all biopsies were processed for both staining methods.

**Table 3:** Logistic regression predicting likelihood of a positive biopsy result based on 5 signs and symptoms.

	*B*	S.E.	Wald	*df*	*P* value	Odds ratio	95% C.I. for odds ratio	
							Lower	Upper
Abdominal distension	1.408	.556	6.398	1	.011*	4.086	1.373	12.160
Vomiting	1.133	.678	2.788	1	.095	3.104	.821	11.736
Failure to thrive	−.420	1.002	.176	1	.675	.657	.092	4.680
Anal stenosis	−1.038	.824	1.587	1	.208	.354	.070	1.781
Soiling	−2.692	1.189	5.131	1	.024*	.068	.007	.696

Model’s Nagelkerke R^2^ = 0.28, p = 0.003, χ^2^ (5) = 17.96.

*Statistically significant, *P *< .05.

## Author contributions

**Data curation:** Hiba Hadadin, Yazan Hijazein, Laith Hijazein, Sarah Obiedat, Yazeed Hadadin.

**Formal analysis:** Hiba Hadadin, Yazan Hijazein, Yazeed Hadadin.

**Investigation:** Hiba Hadadin, Abdelrahman Obiedat.

**Methodology:** Hebah Tawfiq Daradkeh.

**Writing – original draft:** Hebah Tawfiq Daradkeh.

**Writing – review & editing:** Raed Al-Taher, Hebah Tawfiq Daradkeh, Abdel rahman Al Manasra, Hamza Alduraidi, Malik Juweid.
